# Antiviral Effectivity of Favipiravir Against Peste Des Petits Ruminants Virus Is Mediated by the JAK/STAT and PI3K/AKT Pathways

**DOI:** 10.3389/fvets.2021.722840

**Published:** 2021-09-06

**Authors:** Weifeng Zhang, Hualong Deng, Yanfen Liu, Shaohong Chen, You Liu, Yuntao Zhao

**Affiliations:** ^1^Department of Animal Science, College of Coastal Agricultural Science, Guangdong Ocean University, Zhanjiang, China; ^2^Department of Bioengineering, College of Food Science and Technology, Guangdong Ocean University, Zhanjiang, China

**Keywords:** peste des petits ruminants virus, favipiravir, antiviral activity, signal pathway, ruminants

## Abstract

Peste des petits ruminants virus (PPRV), belonging to the genus *Morbillivirus* in the family *Paramyxoviridae*, causes severe infectious disease in small ruminants and has been rapidly spreading in many parts of Africa, the Middle East, and Asia. Although vaccination is considered to be an effective means of controlling PPR, the heat-sensitive nature of the vaccines against PPRV greatly limits their application in areas with a hot climate. In the present study, we investigated the anti-PPRV effects of favipiravir and sought to identify the underlying mechanisms *in vitro* using the Vero cell line. MTT assays, Western blotting, indirect immunofluorescence assays, virus plaque formation assays, and qRT-PCR were used to assess the effects of favipiravir on the life cycle of PPRV and the expression of RNA-dependent RNA polymerase (RdRp). Additionally, the expression levels of JAK1, STAT1, phosphorylated (p)-STAT1, PI3K, AKT, and p-AKT, as well as those of signaling molecules acting downstream of the JAK/STAT and PI3K/AKT signaling pathways, were determined by Western blotting and qRT-PCR. The results indicated that, in PPRV-infected, favipiravir-treated Vero cells, the attachment, invasion, replication, and release of PPRV were significantly inhibited, as was the expression of RdRp, when compared with that in untreated PPRV-infected cells. Furthermore, in favipiravir-treated cells, the expression of JAK1 and STAT1 was downregulated, whereas that of p-STAT1 was significantly upregulated. Similarly, the expression levels of PKR, IRF9, ISG54, and MxA proteins that are associated with innate antiviral activity in host cells were also markedly increased. Moreover, with favipiravir treatment, the expression of PI3K and p-AKT and the p-AKT/AKT ratio were significantly decreased, whereas the expression of AKT was noticeably upregulated. The expression of GSK3, NF-κB p65, p-NF-κB p65, and BAD was also increased with favipiravir treatment, while the expression of CREB, p-CREB, p-GSK3, and Bcl-2 was slightly decreased. In addition, all the p-GSK3/GSK3, p-CREB/CREB, p-NF-κB/NF-κB, and p-BAD/BAD ratios were significantly reduced in favipiravir-treated cells. These results implied that the antiviral effectivity of favipiravir against PPRV is mediated by the JAK/STAT and PI3K/AKT pathways and that favipiravir has potential for use as an effective antiviral agent against PPRV.

## Introduction

Peste des petits ruminants (PPR) is a febrile, highly contagious, and often fatal disease, characterized by fever, mucopurulent secretions from the eyes and nose, necrotic stomatitis, bronchopneumonia, and necrohemorrhagic enteritis (1). The causative agent, PPR virus (PPRV), is a representative member of the genus *Morbillivirus* in the family *Paramyxoviridae* (2). PPRV infection leads to almost 100% morbidity and up to 90% mortality, particularly in goats and sheep, resulting in serious economic losses (3). Vaccination is considered as an effective means of controlling PPR. Accordingly, the World Organisation for Animal Health and the Food and Agriculture Organization of the United Nations have undertaken a global vaccination program for the control and eradication of this disease (4).

Generally, the scientific findings on the mechanism underlying the immunosuppressive effects induced by PPRV infection are based on comparisons with related morbilliviruses, such as rinderpest virus (RPV), measles virus (MV), and canine distemper virus (CDV) (2). Studies have shown that morbilliviruses display a pronounced tropism for lymphoid tissues and cause extensive damage to lymphoid organs, leading to immunosuppression (5). The non-structural protein V of morbilliviruses plays an important role in interferon (IFN)-mediated immune responses, especially those associated with type I IFNs (IFNα/IFNβ), which are responsible for the innate antiviral effects of host cells. Immunosuppression may result from the inhibition of IFN synthesis, IFN-induced antiviral proteins, and immunoglobulin production, which finally blocks IFN signal transduction (6). In addition, the N protein of morbilliviruses has been found to inhibit T lymphocyte proliferation and the functions of dendritic cells in mice, thereby affecting cell-mediated immune responses (7). Moreover, the N protein can bind to the type II receptor for the Fc region (FcγRII) of immunoglobulin G on human and murine B lymphocytes and inhibit antibody production *in vitro* (8). These strategies allow morbilliviruses to evade the innate immune system and propagate, thereby increasing disease severity.

Live attenuated vaccines have been used to control PPR in different endemic areas of the world. The commonly used vaccine strains Nigeria 75/1 and Sungri/96 are recognized as being safe and effective and do not exert significant immunosuppressive effects (9, 10). However, subclinical or sporadic cases of PPR still occur in some inoculated animals due to poor protective efficacy of the vaccine. The few survivors may develop secondary infections with other opportunistic pathogens such as *Pasteurella* spp., *Escherichia coli*, and *Mycoplasma* spp. owing to an impaired immune system (7, 10).

To date, no antiviral compound has been approved for the treatment of PPRV-infected animals, and relatively few studies have been undertaken either *in vitro* or *in vivo* with the aim of developing antiviral agents against PPRV. As no vaccination alone can provide instantaneous protection, it is important that antiviral compounds are identified that can be administered before virus-specific neutralizing antibodies and cell-mediated response are induced in vaccinated animals, thereby helping to control the spread of the virus. A recent study demonstrated that favipiravir, a competitive inhibitor of RNA-dependent RNA polymerase (RdRp), significantly inhibits the replication of CDV *in vitro* in a dose-dependent manner (11). Here, we evaluated the potential anti-PPRV efficiency of favipiravir *in vitro* and systematically investigated its mechanism of action.

## Materials and Methods

### Cells, Virus, Antibodies, and Compounds

Vero cells were procured from the Cell Bank of the Chinese Academy of Sciences (Shanghai, China) and grown in essential Dulbecco's modified Eagle's medium (DMEM; Gibco, Grand Island, NY, USA) supplemented with 10% fetal bovine serum (FBS) (Gibco), 100 IU/ml of penicillin, and 10 μg/ml of streptomycin (Sigma-Aldrich, St. Louis, MO, USA) at 37°C with 5% CO_2_.

Live attenuated PPRV vaccine strain Nigeria 75/1 was obtained from the China Institute of Veterinary Drug Control (Beijing, China). PPRV was inoculated into Vero cells and cultured in DMEM supplemented with 2% FBS at 37°C with 5% CO_2_. Viral titers were estimated using the Reed–Muench method and were expressed as 50% tissue culture infective doses (TCID_50_)/ml. The virus suspension was stored at −80°C.

Antibodies targeting B-cell lymphoma 2 (Bcl-2, 7382), glycogen synthase kinase 3 (GSK3, 7291), phosphorylated (p)-GSK3 (3738), cAMP response element binding protein (CREB, 377154), p-CREB (81486), Bcl-2-associated agonist of cell death (BAD, 8044), and p-BAD (271963) were obtained from Santa Cruz Biotechnology, Inc. (Paso Robles, CA, USA). Antibodies against Janus kinase 1 (JAK1, 3716), signal transducer and activator of transcription 1 (STAT1, 6772), p-STAT1 (6772), phosphatidylinositol 3-kinase (PI3K, 4249), protein kinase B (AKT, 4691), p-AKT (4060), nuclear factor kappa B (NF-κB) p65 (6956), and p-NF-κB p65 (3033) were purchased from Cell Signaling Technology (Danvers, MA, USA). The polyclonal antibodies against the H, F, and N proteins of PPRV were raised as previously described (12).

Favipiravir (6-fluoro-3-hydroxy-2-pyrazinecarboxamide) was purchased from Beijing Fan De Biotechnology Company (Beijing, China). Favipiravir was dissolved in dimethyl sulfoxide (DMSO), diluted to 100 mg/ml with essential DMEM medium, and stored at −80°C. For the experiments, favipiravir was diluted to the required concentration in maintenance medium.

### Assessment of Favipiravir Cytotoxicity

The cytotoxicity of the compound was assessed by MTT assay as previously described (13). Briefly, Vero cells were seeded in 96-well plates (1 × 10^4^ cells/well) and incubated at 37°C with 5% CO_2_. At confluence, the cells were incubated with varying concentrations of favipiravir (two-fold dilutions from a starting concentration of 100 μg/ml) for 48 h at 37°C. MTT reagent (20 μl) was then added to each well, and the cells were further incubated at 37°C for 4 h. Then, 150 μl of DMSO was added for solubilization, and the cells were incubated for another 10 min with shaking. Finally, the optical density (OD) of each well at 490 nm was determined using an ELISA microplate reader (BioTek, Winooski, VT, USA).

### The Antiviral Effect of Favipiravir

Vero cells were seeded in 96-well plates (1 × 10^4^ cells/well) and cultured for 24 h at 37°C with 5% CO_2_. After the medium was removed, 100 μl of favipiravir at maximum noncytotoxic concentration and 100 μl of a PPRV suspension at 100 TCID_50_ were simultaneously added to each well-followed by incubation at 37°C. The cell and virus-only (PPRV-infected) controls were set up at the same time. The cytopathic effect of favipiravir on the cells was continuously monitored. Cell viability was detected by MTT assay when the cytopathic effect in the virus-only control group had reached 50%, as previously described (14).

### Immunofluorescence Assay

Vero cells were seeded on coverslips placed at the bottom of each well of a six-well plate and incubated at 37°C until confluence. The cells treated with the PPRV and favipiravir combination were cultured at 37°C, with cells treated with PPRV alone serving as the virus-only control. At the 50% cytopathic effect, the coverslips were removed, the cells were fixed in 4% paraformaldehyde for 30 min, permeabilized with 0.1% Triton X-100 in phosphate-buffered saline (PBS) for 10 min, blocked with 5% bovine serum albumin (BSA) for 1 h, and incubated with anti-PPRV serum at 4°C overnight and then with fluorochrome-conjugated secondary antibody at room temperature for 1 h. Finally, the cell nuclei were counterstained with 4′,6-diamidino-2-phenylindole (DAPI) and examined with a fluorescence microscope (Leica, Wetzlar, Germany), as previously described (15).

### Western Blotting

To determine the anti-PPRV efficiency of favipiravir, Vero cells were seeded in cell culture dishes and incubated at 37°C until confluence. PPRV at 100 TCID_50_ and favipiravir at the final concentration of 50 μg/ml were then added to the cells, with those treated with PPRV alone serving as the virus-only control. When the cytopathic effect had reached 50% in the control group, the cells were harvested, and the protein was extracted using radioimmunoprecipitation assay (RIPA) buffer (Beyotime, Shanghai, China). Protein concentrations were determined using the bicinchoninic acid (BCA) method. Equal amounts of protein were separated by 10% sodium dodecyl sulfate–polyacrylamide gel electrophoresis (SDS–PAGE), transferred onto a polyvinylidene difluoride membrane (Millipore, Billerica, MA, USA), blocked with 5% skimmed milk for 1 h, and incubated overnight with primary antibody at 4°C and then with horseradish peroxidase (HRP)-conjugated secondary antibody for 1 h. The bands were visualized using chemiluminescence, as previously described (16).

### Quantitative Real-Time Reverse Transcription–Polymerase Chain Reaction

To evaluate the effect of favipiravir on PPRV propagation, Vero cells at 60% confluence were treated with a combination of favipiravir at the final concentration of 50 μg/ml and PPRV at 100 TCID_50_ and incubated for 48 h at 37°C with 5% CO_2_. Total RNA was then extracted from the cells with TRIzol reagent (TransGen, Beijing, China), the concentration was measured using an Epoch microplate reader (BioTek), and 1 μg of RNA was used for reverse transcription using the All-In-One RT SuperMix Kit (Vazyme, Nanjing, China) following the manufacturer's instructions. The reaction mixture for qPCR consisted of 2 μl of cDNA, 10 μM of primers, and 10 μl of SYBR qPCR Master Mix in a total volume of 20 μl. The cycling conditions were 95°C for 30 s followed by 40 cycles of 95°C for 5 s and 60°C for 30 s. The expression levels of the genes coding for PPRV structural proteins and RdRp were normalized to those of GAPDH and quantified using the 2^−ΔΔCT^ method. The primer sequences are shown in Table 1.

**Table 1:** Sequences of primers used for qRT-PCR.

**Genes**	**Sequence (5′ → 3′)**
*PPRV(N)-F*	CTCGGAAATCGCACAGAC
*PPRV(N)-R*	TCTTCTCTGGTCGCTGGT
*PPRV(H)-F*	ATGGTTGTATTGCCGACGAAGGAC
*PPRV(H)-R*	GAGGAACTTAATCTTATCG
*RdRp-F*	AGGGATGCTGCTCGGTCTTGG
*RdRp-R*	CGTGTAGGTGTAGCACTGTGG
*PKR-F*	ATAGCAAGAAGGCAGAGCGTGAAG
*PKR-R*	TCAAGTCCATCCCAACAGCCATTG
*IRF9-F*	GACTACTCACTGCTGCTCACCTTC
*IRF9-R*	GCTCCATGCTGCTCTCAGAACC
*ISG20-F*	ATGGACTGCGAGATGGTGGG
*ISG20-R*	CCCTCAGGCCGGATGAACTT
*ISG54-F*	CCAACCAAGCAAGTGTGAGGAGTC
*ISG54-R*	CTTCTGCCAGTCTGCCCATGTG
*MxA-F*	GCATCTCCAGCCACATCCCTTTG
*MxA-R*	TGGTGTCGCTCCGCTCCTTC
*GADPH-F*	CATGACCACAGTCCACGCCATC
*GADPH-R*	GATGACCTTGCCCACAGCCTTG

### The Effect of Favipiravir on Peste Des Petits Ruminants Virus Attachment to Vero Cells

Immunofluorescence assays (IFAs) and qRT-PCR were used to evaluate the effect of favipiravir on PPRV attachment to Vero cells. Vero cells seeded on coverslips were treated with PPRV and favipiravir and subsequently examined by IFA, as mentioned above (17). Simultaneously, confluent Vero cells were pretreated with favipiravir at 37°C for 1 h in a six-well plate and then infected with 100 TCID_50_ PPRV at 4°C for 2 h. The cells were harvested, washed five times with PBS, and then subjected to qRT-PCR for the measurement of the expression levels of PPRV genes.

### The Effect of Favipiravir on Peste Des Petits Ruminants Virus Entry into Vero Cells

As mentioned above (18), confluent Vero cells were infected with 100 TCID_50_ PPRV in a six-well plate at 4°C for 1 h, washed five times with cold PBS, treated with favipiravir at 37°C for 1 h, washed again with PBS to remove any extracellular virus, and then incubated with cell culture medium for 36, 48, and 60 h. The levels of the viral structural proteins hemagglutinin protein (H), fusion protein (F), and nucleocapsid protein (N) in cell lysates were then assessed by Western blotting.

### The Effect of Favipiravir on the Release of Peste Des Petits Ruminants Virus Progeny

Vero cells were infected with 100 TCID_50_ PPRV at 37°C for 48 h, washed five times with cold PBS, and then treated with favipiravir at a final concentration of 50 μg/ml for 2 h. The numbers of progeny virus particles released in the supernatants were assessed using a plaque formation assay, as previously described (19, 20).

### Statistical Analysis

The data were analyzed using GraphPad Prism 5 (GraphPad Prism Software, La Jolla, CA, USA) and were expressed as means ± standard deviation. Differences between two groups were evaluated by Student's *t*-test, while differences among multiple groups were compared by one-way analysis of variance (ANOVA). A *p*-value <0.05 was considered statistically significant.

## Results

### The Cytotoxic Effects of Favipiravir on Vero Cells

**Figure 1:**
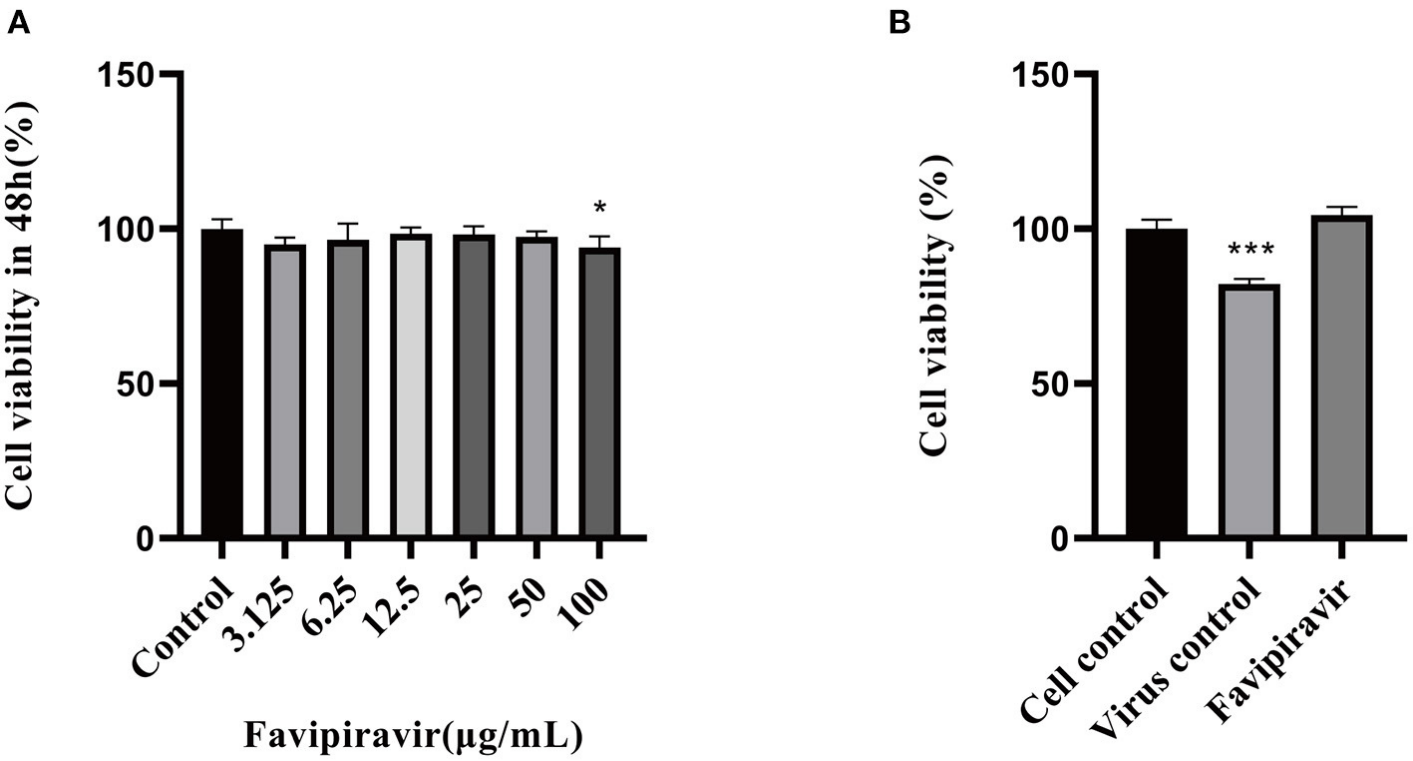
Determination of the maximal noncytotoxic concentration of favipiravir. **(A)** The viability of Vero cells treated with serial dilutions of favipiravir for 48 h from a starting concentration of 100 μg/ml. **(B)** The viability of Vero cells treated with 100 TCID_50_ peste des petits ruminants virus (PPRV) and favipiravir at maximal non-toxic concentration for 48 h (*p < 0.05, ***p < 0.001 vs. the cell control).

The cytotoxic effects of favipiravir on Vero cells were detected by MTT assay. As shown in Figure 1A, the maximal non-cytotoxic concentration of favipiravir was 50 μg/ml.

### The Antiviral Effectivity of Favipiravir Against Peste Des Petits Ruminants Virus *in vitro*

The viability of PPRV-infected cells treated with favipiravir at 50 μg/ml for 48 h was significantly higher than that of untreated PPRV-infected cells (virus-only controls). In the latter, the cytopathic effect reached 50%, and cell viability was significantly decreased in the virus-only control group (Figure 1B).

**Figure 2:**
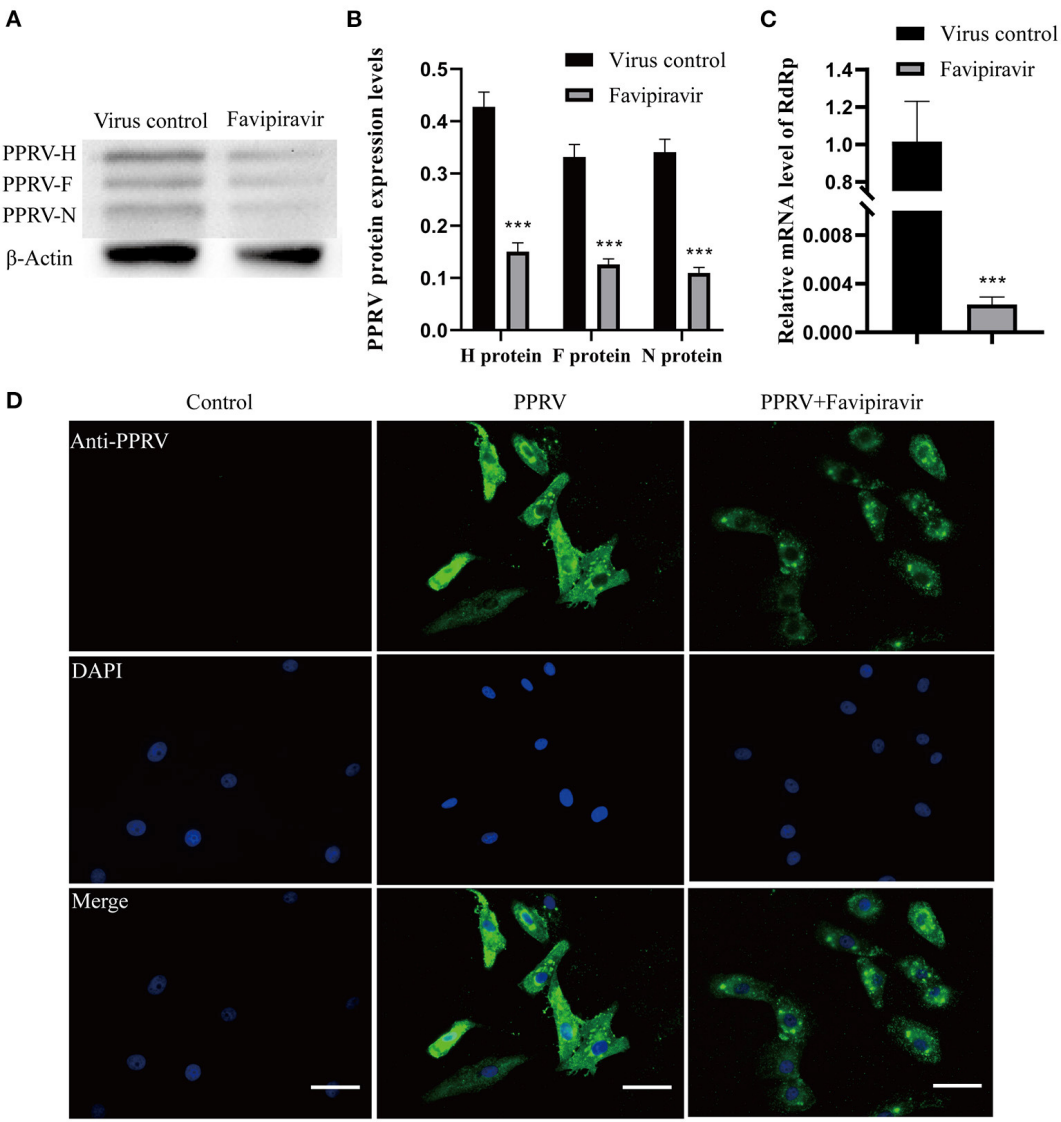
The anti-peste des petits ruminants virus (anti-PPRV) effect of favipiravir in Vero cells. **(A,B)** Vero cells were treated with PPRV at 100 TCID_50_ and favipiravir at 50 μg/ml for 48 h, following which the expression of the structural proteins H, F, and N was analyzed by Western blotting (***p < 0.001 vs. the virus-only control). **(C)** Vero cells were treated with favipiravir and PPRV simultaneously for 48 h, and then the relative mRNA expression levels of the H and N genes were measured by qRT-PCR (***p < 0.001 vs. the virus-only control). **(D)** The anti-PPRV activity of favipiravir was detected by immunofluorescence assay (IFA) in PPRV-infected Vero cells after 48 h. PPRV is in green; nuclei are stained blue (DAPI). Scale bars = 50 μm.

The Western blotting analysis showed that the expression levels of the PPRV structural proteins H, F, and N were significantly lower in PPRV-infected, favipiravir-treated cells than in the virus-only controls (Figures 2A,B). Moreover, favipiravir treatment significantly reduced the expression levels of RdRp as determined by qRT-PCR (Figure 2C). Meanwhile, analysis using IFA indicated that substantially fewer virions were distributed in the cytoplasm of PPRV-infected, favipiravir-treated Vero cells when compared with control cells. Compared with untreated PPRV-infected cells, few syncytia were observed in PPRV-infected, favipiravir-treated cells, suggesting that favipiravir could inhibit PPRV propagation in Vero cells (Figure 2D) and alleviate the cytopathic effect induced by virus infection.

### The Effect of Favipiravir on Different Phases of the Peste Des Petits Ruminants Virus Life Cycle

**Figure 3:**
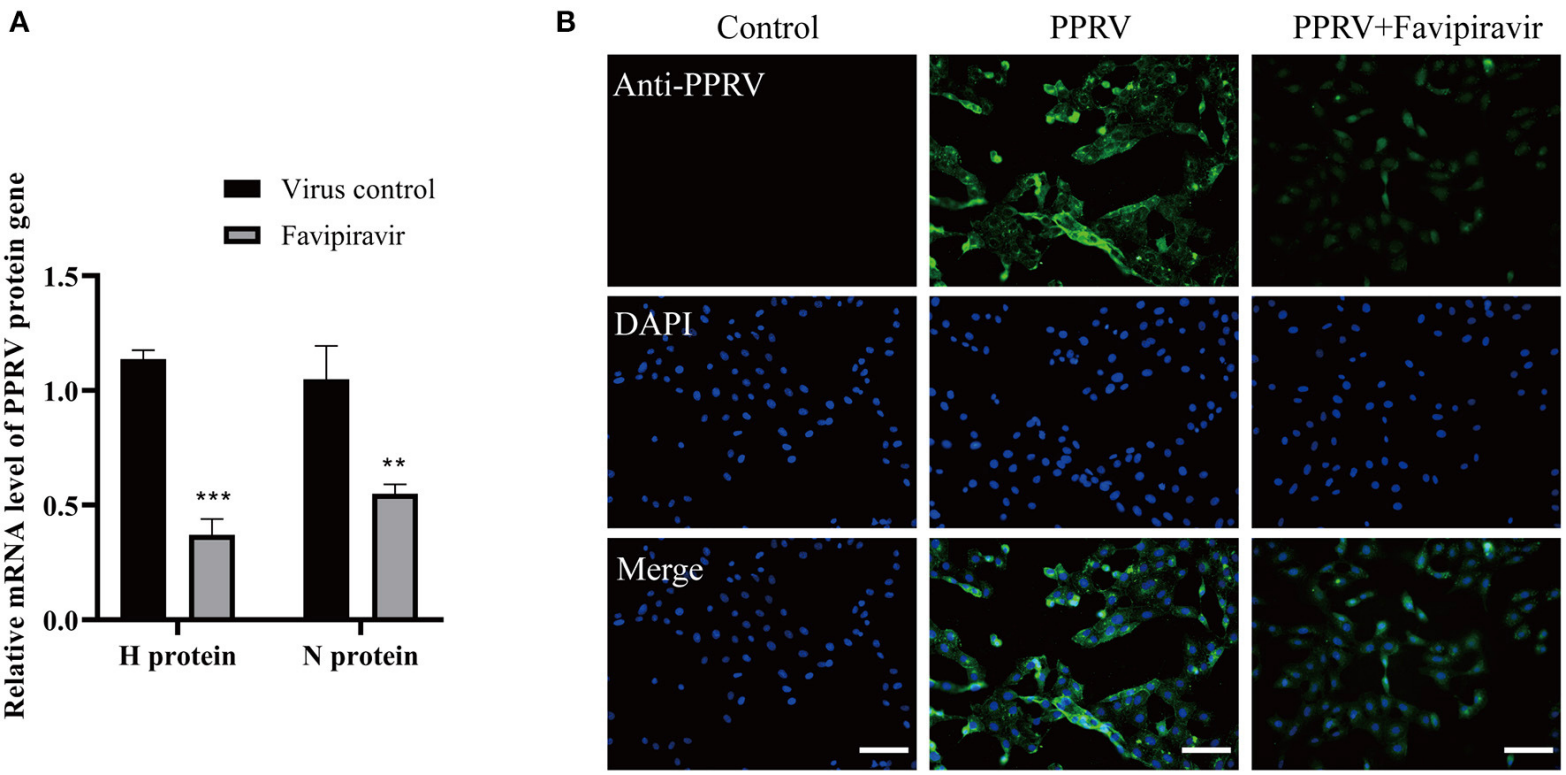
The effect of favipiravir on peste des petits ruminants virus (PPRV) attachment to host cells. Vero cells were pre-incubated with favipiravir at 50 μg/ml or an equal volume of medium at 37°C for 1 h and then infected with PPRV at 4°C for 2 h. The cells were washed five times with cold phosphate-buffered saline (PBS) and collected for qPCR and immunofluorescence assay (IFA) detection (**p < 0.01, ***p < 0.001 *vs*. the virus-only control). **(A)** The mRNA expression of the H and N genes. **(B)** PPRV distribution in the cytoplasm was detected by indirect IFA. Scale bars = 100 μm.

Compared with those in the virus-only controls, the expression levels of the H and N genes of PPRV were significantly decreased in PPRV-infected, favipiravir-treated Vero cells (Figure 3A). The IFA results also showed that the numbers of PPRV virions distributed in the cytoplasm were significantly reduced with favipiravir treatment and that most of the infected cells displayed a normal morphology. In contrast, round and fused cells could be seen among the untreated controls (Figure 3B). These results suggested that favipiravir can significantly limit the attachment of PPRV to Vero cells.

**Figure 4:**
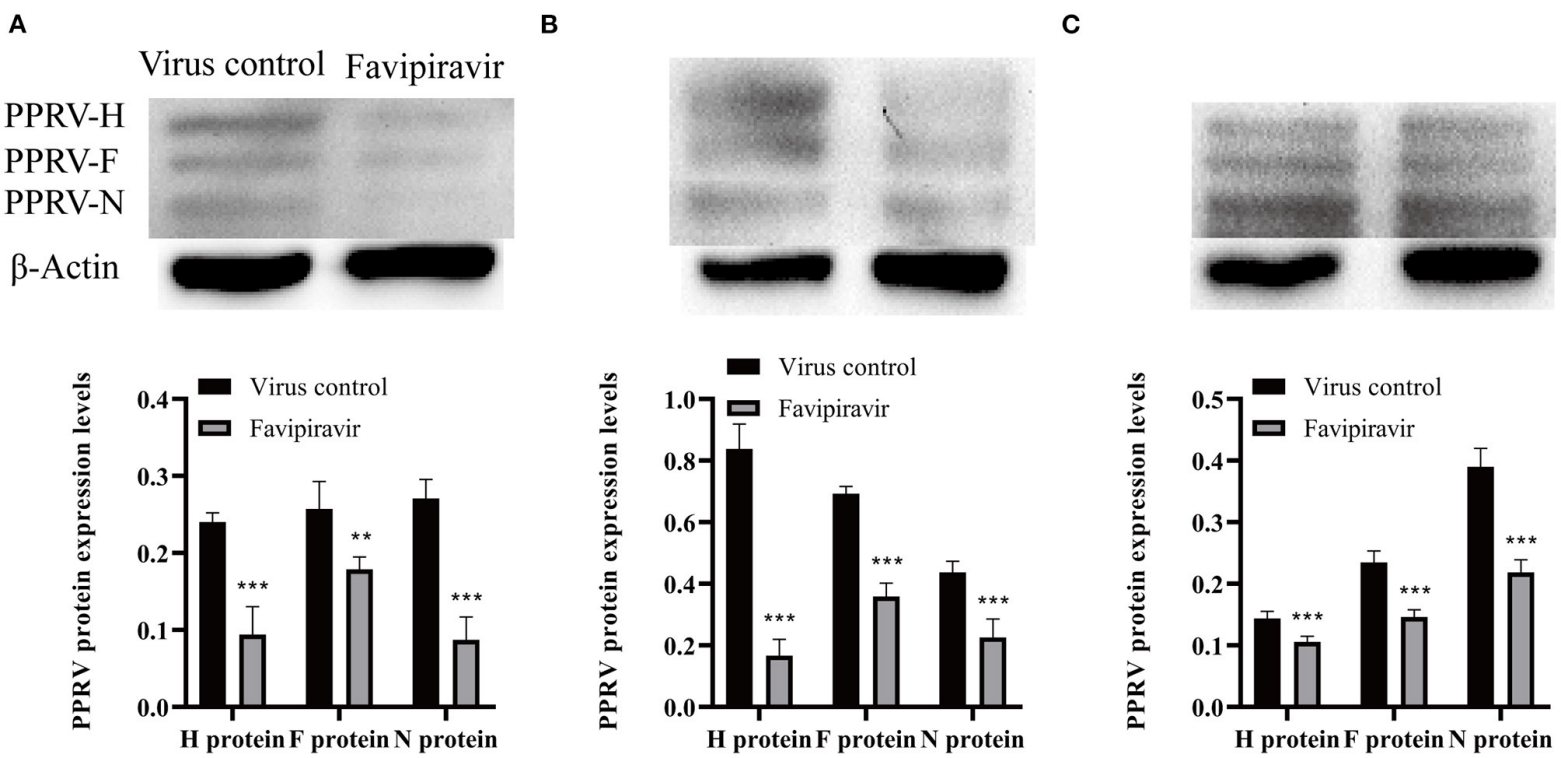
Vero cells were infected with peste des petits ruminants virus (PPRV) at 100 TCID_50_/ml at 4°C for 1 h, washed with cold phosphate-buffered saline (PBS) to remove unattached viruses, and then treated with favipiravir at 50 μg/ml in maintenance medium supplemented with 2% fetal calf serum (FCS) for 1 h at 37°C. The cells were then washed again with PBS to remove any extracellular viruses, and maintenance medium was added. The plates were incubated at 37°C, and the expression of the viral structural proteins H, F, and N was analyzed by Western blotting at **(A)** 36, **(B)** 48, and **(C)** 60 h post-invasion (**p < 0.01, ***p < 0.001 vs. the virus-only control).

The expression levels of the PPRV H, F, and N structural proteins were significantly decreased at 36, 48, and 60 h post-invasion in favipiravir-treated Vero cells (Figure 4), implying that favipiravir treatment could block PPRV entry into host cells. Moreover, the expression levels of the viral structural proteins showed significant increases with prolonged invasion time and tended to approach those seen in untreated virus-infected cells (controls). These findings suggested that early favipiravir administration can effectively block PPRV entry into host cells.

**Figure 5:**
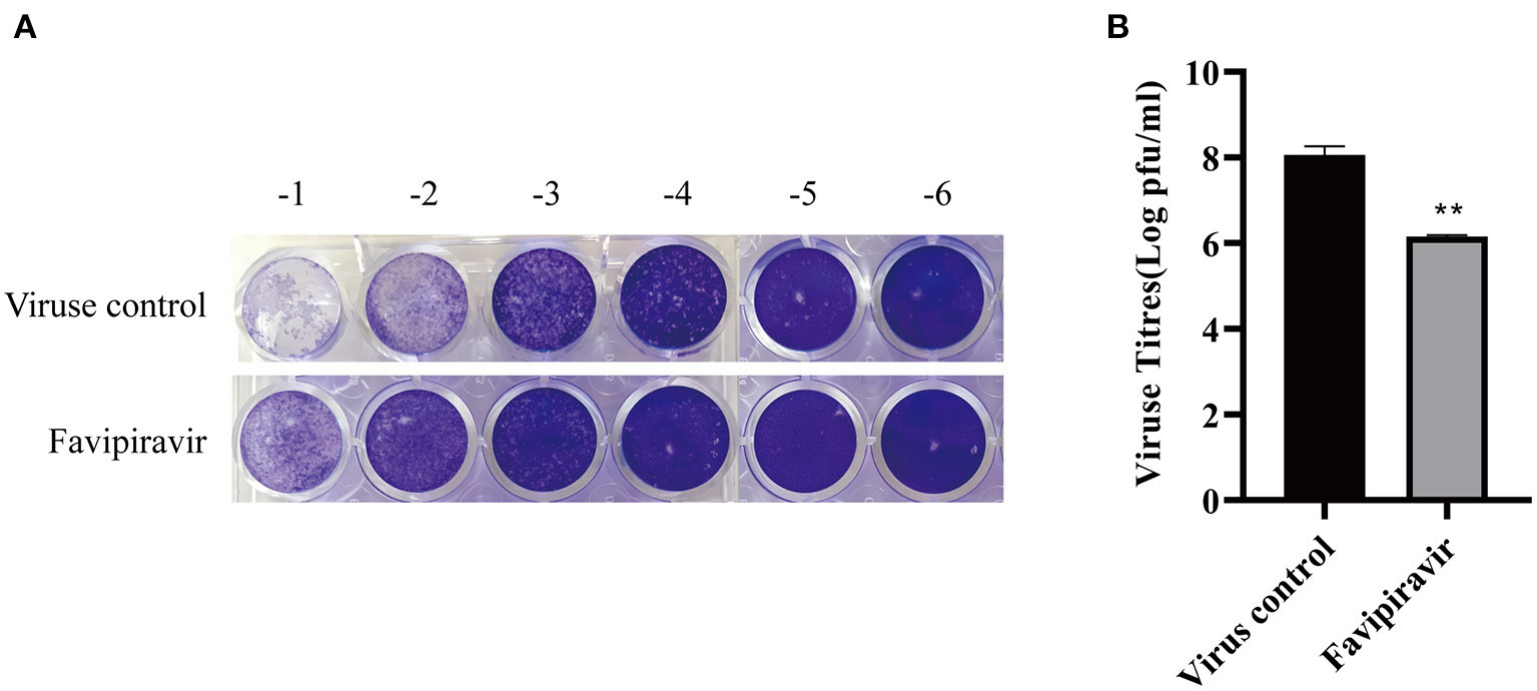
The effect of favipiravir on the release of progeny peste des petits ruminants virus (PPRV) particles from host cells. **(A,B)** Vero cells were infected with 100 TCID_50_ PPRV at 37°C for 2 h and washed five times with cold phosphate-buffered saline (PBS) to remove unattached viruses, followed by supplementation with maintenance medium. The cells were incubated at 37°C for 48 h and washed five times with cold PBS. Finally, the cells were incubated with maintenance medium containing favipiravir at 50 μg/ml for 2 h. The supernatants were collected, and the virus titers were determined by plaque formation assay (**p < 0.01 vs. the virus-only control).

The effect of favipiravir on the release of PPRV progeny from infected Vero cells was assessed using a plaque formation assay. The results showed that PPRV titers released from PPRV-infected, favipiravir-treated cells were significantly lower than those released from untreated PPRV-infected cells (Figures 5A,B), indicating that favipiravir could effectively inhibit PPRV release from host cells.

### The Anti-Peste Des Petits Ruminants Virus Activities of Favipiravir Are Mediated by the JAK/STAT and PI3K/AKT Signaling Pathways

**Figure 6:**
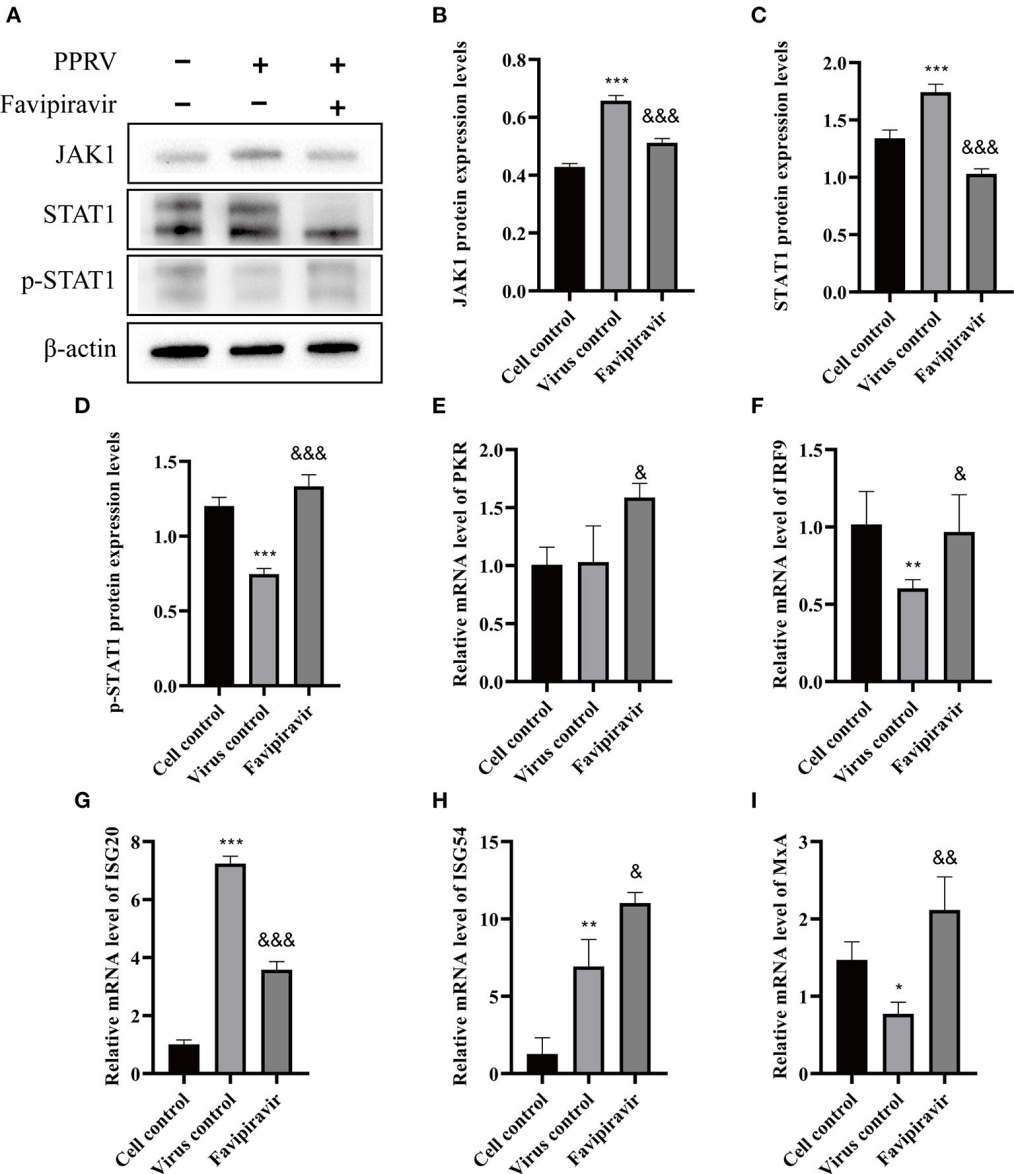
The effect of favipiravir on the expression levels of proteins and mRNAs related to the JAK/STAT signaling pathway **(A–I)**. Vero cells were simultaneously treated with favipiravir and infected with peste des petits ruminants virus (PPRV), and protein expression levels were measured 48 h after infection. The detection of JAK1 **(B)**, STAT1 **(C)**, and p-STAT1 **(D)** expression levels by Western blotting. The relative mRNA levels of PKR **(E)**, IRF9 **(F)**, ISG20 **(G)**, ISG54 **(H)**, and MxA **(I)** were detected by qRT-PCR (^&^*p* < 0.05, ^&&^*p* < 0.01, ^&&&^*p* < 0.001 vs. the virus-only control; *p < 0.05, **p < 0.01, ***p < 0.001 vs. the cell control).

To further elucidate the molecular mechanisms underlying the antiviral effects of favipiravir, we assessed the expression levels of key proteins involved in the JAK/STAT and PI3K/AKT signaling pathways. Western blotting results revealed that the JAK1 and STAT1 protein levels were significantly downregulated in PPRV-infected cells treated with favipiravir and that the level of p-STAT1 was significantly increased compared with that seen in control (untreated, PPRV-infected) cells (Figures 6A–D). These observations implied that the JAK/STAT signaling pathway was inhibited in PPRV-infected cells, an effect that could be reversed with favipiravir treatment.

The mRNA levels of double-stranded RNA-dependent protein kinase (PKR), IFN regulatory factor 9 (IRF9), IFN-stimulated gene 54 (ISG54), and myxovirus resistance protein A (MxA), signaling molecules that are closely related to innate antiviral activity in host cells, were markedly upregulated in PPRV-infected, favipiravir-treated cells when compared with those of control, untreated virus-infected cells, whereas those of ISG20 were significantly downregulated (Figures 6E–I).

**Figure 7:**
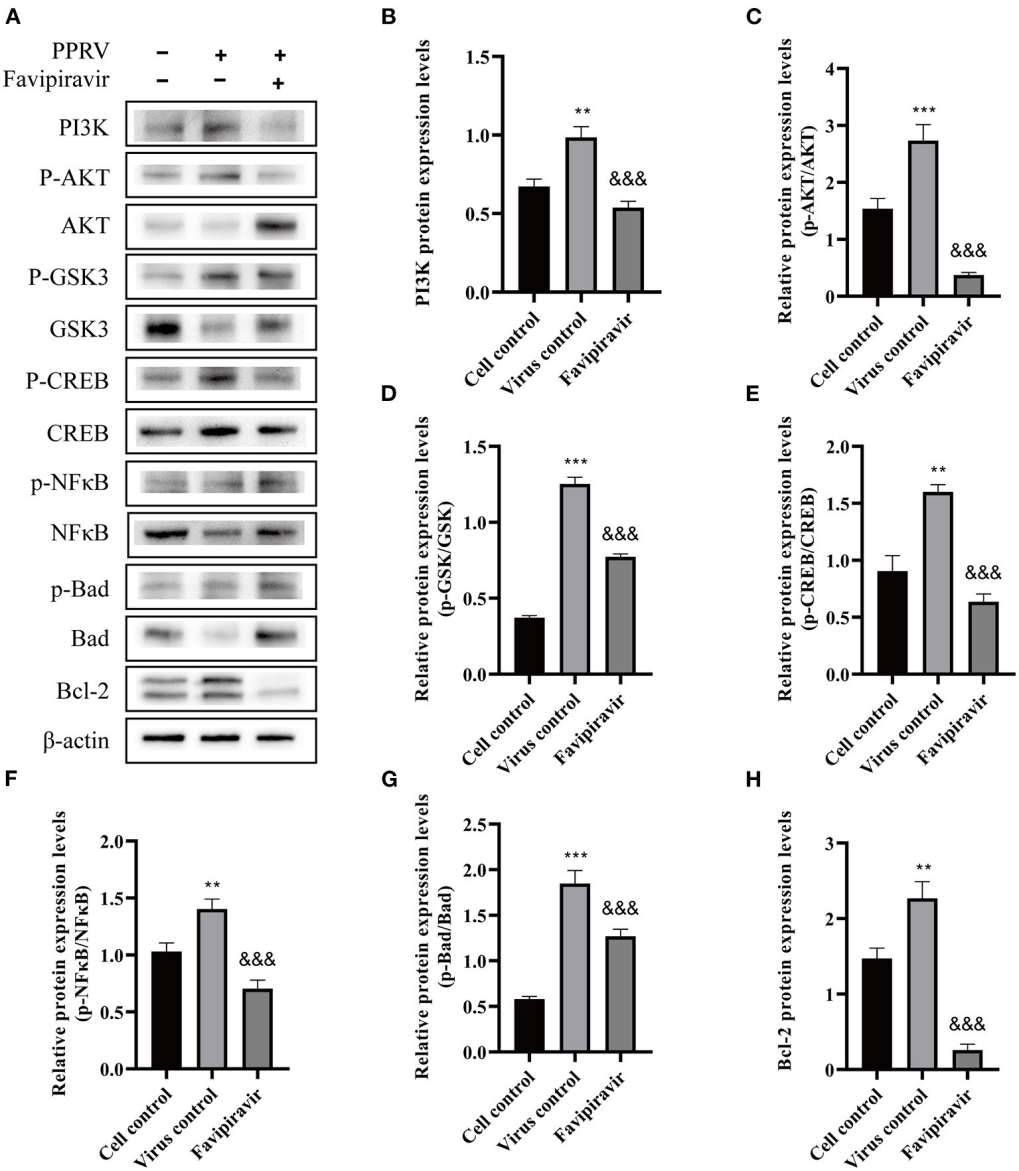
The effects of favipiravir on the expression levels of key proteins related to the PI3K/AKT signaling pathway. Vero cells were simultaneously treated with favipiravir and infected with peste des petits ruminants virus (PPRV); and the expression levels of PI3K, p-AKT, AKT, p-GSK3, GSK3, p-CREB, CREB, p-NF-κB p65, NF-κB p65, p-BAD, BAD, and Bcl-2 **(A–H)** were detected by Western blotting 48 h after infection (^&&&^*p* < 0.001 vs. the virus-only control; **p < 0.01, ***p < 0.001 vs. the cell control).

Compared with the cell control group (Figure 7A) (untreated, uninfected), the expression of PI3K and p-AKT was significantly upregulated, the p-AKT/AKT ratio was increased, and the expression of AKT was downregulated in untreated PPRV-infected cells (Figures 7B,C); however, the opposite effects were seen with favipiravir treatment.

The expression of GSK3 was slightly downregulated, while that of p-GSK3 was upregulated in untreated PPRV-infected cells. Additionally, compared with that in the virus-only controls, the expression of CREB and p-CREB was downregulated with favipiravir treatment, whereas the NF-κB p65, p-NF-κB p65, BAD, and p-BAD expression levels displayed the opposite tendency. Moreover, the p-GSK3/GSK3, p-CREB/CREB, p-NF-κB p65/NF-κB p65, and p-BAD/BAD ratios were all significantly decreased with favipiravir treatment (Figures 7D–G). Notably, the expression of Bcl-2 was significantly upregulated in PPRV-infected cells (Figure 7H) but was markedly reduced following favipiravir treatment.

Combined, these data suggested that the anti-PPRV activity of favipiravir was mediated by the JAK/STAT and PI3K/AKT signaling pathways. Although PPRV infection inhibited the JAK/STAT pathway, this effect was reversed with favipiravir treatment *via* the upregulation of p-STAT1 expression; the expression levels of antiviral proteins such as PKR, IRF9, ISG54, and MxA were elevated accordingly, which led to enhanced innate immunity. In contrast, favipiravir treatment inhibited the PPRV-induced activation of the PI3K/AKT signaling pathway and markedly altered the expression profile of downstream signaling molecules associated with the regulation of cell apoptosis, thereby blocking persistent PPRV propagation in host cells.

## Discussion

Favipiravir, a recently identified RdRp inhibitor, was approved for use in the treatment of influenza virus infection in Japan in 2014 (21). Favipiravir has been shown to exert antiviral activity against a broad range of paramyxoviruses, including MV, human respirovirus 3, CDV, respiratory syncytial virus (RSV), mumps virus, and human metapneumovirus (21, 22). Studies have demonstrated that favipiravir is converted into its active form, favipiravir-ribofuranosyl-5′-triphosphate (favipiravir-RTP), *in vivo*, and subsequently binds intracellular RdRp (23), leading to the inhibition of virus replication and transcription of the viral genome (24). These findings suggest that favipiravir is a potential antiviral agent against a wide range of RNA viruses; however, the antiviral effectivity of favipiravir against PPRV and the associated mechanism of action have yet to be investigated.

Vaccination against PPRV is widely used as a means of controlling PPR and is recognized as a key tool in the global PPR eradication program (25). However, the live attenuated vaccine is heat sensitive, and an effective cold chain is required to deliver the vaccine in areas with a hot climate, which is costly and inconvenient (26). These observations highlight the need for the development of antiviral agents that can effectively block PPRV transmission from carriers to close contacts; however, there is a paucity of data regarding PPRV-targeting antiviral drugs.

Here, we demonstrated that favipiravir treatment could inhibit PPRV propagation and PPRV infection-mediated cytopathic effects. Our findings showed that favipiravir exerted inhibitory effects against viral attachment, invasion, replication, and release. Recent studies have shown that the methanol extract of *Polyalthia longifolia* leaves can inhibit PPRV invasion and release *in vitro*, while plant extracts containing phytochemicals such as tannins and flavonoids were found to strongly inhibit HIV-1 protease (19). Additionally, silver nanoparticles can reportedly interact with the surface of PPRV, as well as with its core, thereby impairing its entry into host cells, and have also been reported to interact with HIV-1, Tacaribe virus, and herpes simplex virus type 1 (HSV-1) (18). Favipiravir only acts on inhibiting the release of CDV from virus-infected cells, because studies have demonstrated that favipiravir exerts its effects at the early and intermediate stages of viral replication (11). Here, we found that favipiravir impaired PPRV release, possibly by interfering with RdRp, and inhibited PPRV invasion and release, which suggested that favipiravir may block the receptor binding sites on PPRV envelope glycoprotein or interact with the PPRV. Therefore, we speculate that the action mechanism of antivirals may be diverse. Many antiviral agents, such as ribavirin, remdesivir, sofosbuvir, galidesivir, and tenofovir, act by inhibiting RdRp activation (27). For example, remdesivir and favipiravir were shown to be competitive inhibitors of the RdRp of the Ebola virus and the influenza virus, respectively, thereby inhibiting viral RNA synthesis (28, 29). Our results indicated that favipiravir inhibited PPRV propagation by decreasing RdRp expression, which was consistent with previous reports.

Innate immunity represents an adaptive immune response to the presence of a variety of pathogenic microorganisms or antigens (30, 31). During viral infection, pathogens can be recognized by pathogen-recognition receptors in host cells (31), leading to the activation of the JAK/STAT signaling pathway and the subsequent induction of genes containing IFN-stimulated response elements (ISREs) (32). To escape from the IFN-mediated antiviral responses of host cells, viruses have developed different strategies to inhibit the activation of this pathway through their viral proteins (33). STAT proteins play a key role in IFN signal transduction. Paramyxoviruses can inhibit IFN activation, while CDV, simian virus type 5 (SV5), and RPV can all suppress the activity of p-STAT1 (34). PPRV can also inhibit the induction of IFNs. The V protein of PPRV can interact with JAK1, TYK2, and STAT1/2; block STAT1/2 nuclear translocation; and inhibit the phosphorylation of IFN regulatory factor 3 (IRF3) (16). Furthermore, the V, C, and P proteins of PPRV can also bind to p-STAT1, which inhibits STAT1 activity; the N protein can block IRF3 nuclear translocation; and the N/P protein combination can inhibit IFN binding to ISREs, thereby affecting the expression of ISGs (16, 35, 36).

In this study, the JAK/STAT signaling pathway was inhibited in PPRV-infected cells, whereas STAT1 phosphorylation levels were significantly increased with favipiravir treatment, indicating that the JAK/STAT pathway had been reactivated. Accordingly, the mRNA expression levels of PKR, IRF9, ISG54, and MxA, downstream signaling molecules associated with innate antiviral immunity of the JAK/STAT pathway, were markedly upregulated in PPRV-infected, favipiravir-treated cells. Hence, these results indicated that the anti-PPRV effect of favipiravir is mediated *via* the JAK/STAT pathway.

The PI3K/AKT signaling pathway is closely related to cell growth, survival, metabolism, and proliferation. An increasing number of studies have reported that high expression levels of signaling molecules downstream of the PI3K/AKT pathway are conducive to viral survival and proliferation (37). For example, the PI3K/AKT signaling pathway is activated in the early stages of Ebola virus infection, which promotes the endocytosis of the virus (38). The activation of this pathway involves the recruitment of PI3K to the cell membrane, where it combines with the phosphoinositides phosphatidylinositol-3,4,5-trisphosphate (PIP3) and phosphatidylinositol-3,4-bisphosphate (PIP2), leading to the recruitment of AKT and its phosphorylation by PIP3 (39). Several studies have shown that Marek's disease virus (MDV) and porcine epidemic diarrhea virus (PEDV) can promote their own replication by activating the PI3K/AKT pathway (40, 41). Meanwhile, the activity of the AKT protein was decreased by MV, which is similar to PPRV (42). The reduced expression of p-AKT in host cells can inhibit the replication of influenza A virus (IAV) and Newcastle disease virus (NDV) (43, 44), while low PI3K and AKT phosphorylation levels can suppress IAV and HSV-1 propagation, respectively (44, 45). Despite these observations, the mechanism underlying how PPRV regulates PI3K/AKT signaling remains unclear. In the present study, we showed that favipiravir treatment can suppress the PPRV infection-induced activation of the PI3K/AKT signaling pathway. As AKT activation can promote the phosphorylation of downstream factors such as NF-κB, CREB, Bcl-2, BAD, and GSK3 (39), our results indicated that PPRV can modulate the expression levels of molecules that act downstream of PI3K/AKT signaling.

The effects of the phosphorylation of factors downstream of the PI3K/AKT pathway on the regulation of cell apoptosis, metabolism, and proliferation have been well documented (46). CREB and GSK3 are closely associated with cell growth, apoptosis, and immune modulation (46, 47). The phosphorylation of GSK3β at Ser9 and CREB at Ser133 is normally induced by p-AKT; and p-GSK and p-CREB levels were reported to be increased in varicella-zoster virus (VZV)-, dengue virus (DENV)-, HSV-1-, and porcine reproductive and respiratory syndrome virus (PRRSV)-infected cells (48–50). Consistent with these reports, in this study, we found that GSK and CREB phosphorylation levels were also increased in PPRV-infected cells. The NF-κB is a major regulator of inflammation, immunity, and cell survival and is known to be activated during bacterial or viral infections (51). A recent study showed that IAV infection can upregulate the levels of p-NF-κB p65, an effect that was reversed with rhein treatment (52). Pattern recognition receptors (PRRs) can recognize invading viruses and transmit the signal to the Bcl-2 protein (53). Subsequently, the interaction between anti-apoptotic proteins (Bcl-XL, Bcl-2, and Bcl-W) and pro-apoptotic proteins (BAD, Bax, and Bak) is disrupted, resulting in the permeabilization of the mitochondrial membrane and, eventually, cell apoptosis (53, 54). The expression of BAD, a pro-apoptotic Bcl-2 family member, was shown to promote apoptosis in 293T cells and BHK cells infected with Sindbis virus (SNV) (55). Treatment with aloe-emodin or platelet-rich plasma increased the expression of Bcl-2, concomitant with increased BAD phosphorylation levels, which protected infected cells from apoptosis (56, 57). ABT-263, a Bcl-2 inhibitor, has been shown to promote the apoptosis of cells infected with Zika virus (ZIKV), echovirus 1 (EV1) and EV6, Middle East respiratory syndrome coronavirus (MERS-COV), hepatitis B virus (HBV), HSV-1 and HSV-2, and IAV (24, 58). Given these observations, the PI3K/AKT signaling pathway is expected to be an effective target for antiviral therapy.

In the present study, we showed that favipiravir treatment significantly inhibited the PI3K/AKT pathway and markedly altered the expression profile of signaling molecules downstream of this pathway. Compared with untreated, PPRV-infected cells, the expression of PI3K and p-AKT was significantly decreased, as was the p-AKT/AKT ratio, whereas the expression of AKT was noticeably upregulated in PPRV-infected cells treated with favipiravir. Furthermore, the expression of p-GSK3, CREB, p-CREB, and Bcl-2 was significantly downregulated with favipiravir treatment, whereas that of GSK, NF-κB p65, p-NF-κB p65, BAD, and p-BAD was increased, along with the p-GSK3/GSK3, p-CREB/CREB, p-NF-κB p65/NF-κB p65, and p-BAD/BAD ratios. These results implied that the phosphorylation of these downstream signaling molecules plays a crucial role in the survival of PPRV-infected cells.

In summary, we found that favipiravir treatment effectively inhibited PPRV propagation and the cytopathic effects induced by PPRV infection *in vitro*. Favipiravir treatment also increased the levels of p-STAT1, thereby reversing the PPRV infection-induced inhibition of the JAK/STAT pathway, as well as those of the antiviral molecules PKR, IRF9, ISG54, and MxA, which led to improved innate immunity in the host cells. Moreover, favipiravir treatment enhanced the phosphorylation of signaling molecules acting downstream of the PI3K/AKT pathway, thereby promoting the apoptosis of PPRV-infected cells and inhibiting persistent PPRV propagation in host cells. Therefore, these results indicated that favipiravir is a potential therapeutic agent for the treatment of PPR.

## Data Availability Statement

The original contributions presented in the study are included in the article/supplementary material, further inquiries can be directed to the corresponding author/s.

## Author Contributions

YoL and YZ conceived and designed the experiments. WZ, HD, and SC performed experiments. YaL analyzed the data. YoL and WZ wrote the manuscript. All authors read and approved the final manuscript.

## Conflict of Interest

The authors declare that the research was conducted in the absence of any commercial or financial relationships that could be construed as a potential conflict of interest.

## Publisher's Note

All claims expressed in this article are solely those of the authors and do not necessarily represent those of their affiliated organizations, or those of the publisher, the editors and the reviewers. Any product that may be evaluated in this article, or claim that may be made by its manufacturer, is not guaranteed or endorsed by the publisher.
